# West Nile Virus Transmission in Sentinel Chickens and Potential Mosquito Vectors, Senegal River Delta, 2008–2009

**DOI:** 10.3390/ijerph10104718

**Published:** 2013-10-01

**Authors:** Assane Gueye Fall, Amadou Diaïté, Momar Talla Seck, Jérémy Bouyer, Thierry Lefrançois, Nathalie Vachiéry, Rosalie Aprelon, Ousmane Faye, Lassana Konaté, Renaud Lancelot

**Affiliations:** 1 Laboratoire National d’Elevage et de Recherche Vétérinaire, Institut Sénégalais de Recherches Agricoles, Dakar-Hann BP 2057, Senegal; E-Mails: amadoudiaite@hotmail.com (A.D.); mtseck@hotmail.fr (M.T.S.); jeremy.bouyer@cirad.fr (J.B.); 2 Unité Mixte de Recherche Contrôle des Maladies Animales Exotiques et Emergentes (UMR CMAEE), Centre de Coopération Internationale en Recherche Agronomique pour le Développement (CIRAD), Montpellier 34398, France; E-Mails: thierry.lefrancois@cirad.fr (T.L.); renaud.lancelot@cirad.fr (R.L.); 3 UMR 1309 CMAEE, Institut National de la Recherche Agronomique (INRA), Montpellier 34398, France; 4 UMR CMAEE, INRA, Petit Bourg 97170, Guadeloupe, France; E-Mails: nathalie.vachiery@cirad.fr (N.V.); rosalie.aprelon@cirad.fr (R.A.); 5 UMR 1309 CMAEE, INRA, Petit Bourg 97170, Guadeloupe, France; 6 Faculté des Sciences et Techniques, Département de Biologie Animale, Cheikh Anta Diop University, Dakar BP 5005, Senegal; E-Mails: jogomaye@yahoo.fr (O.F.); konatela@yahoo.fr (L.K.)

**Keywords:** West Nile virus, sentinel chicken, mosquito, *Culex*, Senegal River Delta

## Abstract

West Nile virus (WNV) is an arthropod-borne *Flavivirus* usually transmitted to wild birds by *Culex* mosquitoes. Humans and horses are susceptible to WNV but are dead-end hosts. WNV is endemic in Senegal, particularly in the Senegal River Delta. To assess transmission patterns and potential vectors, entomological and sentinel serological was done in Ross Bethio along the River Senegal. Three sentinel henhouses (also used as chicken-baited traps) were set at 100 m, 800 m, and 1,300 m from the river, the latter close to a horse-baited trap. Blood samples were taken from sentinel chickens at 2-week intervals. Seroconversions were observed in sentinel chickens in November and December. Overall, the serological incidence rate was 4.6% with 95% confidence interval (0.9; 8.4) in the sentinel chickens monitored for this study. Based on abundance pattern, *Culex neavei* was the most likely mosquito vector involved in WNV transmission to sentinel chickens, and a potential bridge vector between birds and mammals.

## 1. Introduction

West Nile virus (WNV) is an arbovirus (*Flaviviridae*, *Flavivirus*) mostly transmitted by *Culex* mosquitoes. Resident birds are the main vertebrate hosts for WNV, and migratory birds spread it across and between continents. Humans and horses are dead-end hosts to WNV [1]. The virus is endemic in Africa, including Senegal, where several serological surveys and entomological studies have been implemented. Observed seroprevalence rates in birds, horses and humans were respectively 5.5% (*n* = 422), 78.3% (*n* = 120) and 80% (*n* = 90) in the Ferlo region, close to Senegal River Delta [2,3,4]. No animal or human clinical cases have been reported.

The Djoudj Ornithological National Park (PNOD) is located in the Senegal River Delta. It is a breeding, feeding, and resting site for hundreds of thousands of resident and migratory birds originating from a wide range of geographic origins [5], and possibly harboring different pathogens, including WNV [6]. Several possible mosquito vector species are present in this area [7]. Migratory birds arrive from Europe in October and leave in March–April of the following year [8]. Their arrival often coincides with the maximum flooding of Senegal River (end of rainy season), and also with a high abundance of mosquitoes. Therefore, it might also be a favorable period for WNV transmission.

This study aimed at identifying periods and areas of high WNV-transmission risk in the Senegal River Delta. For this purpose, we implemented surveys to estimate the population dynamics of potential WNV vectors, together with WNV serological incidence in sentinel chickens (*Gallus gallus domesticus*).

## 2. Material and Methods

The study was conducted in the vicinity of Ross Bethio, close to the Grand Lampsar River—one of the major streams of the Senegal River Delta. This site is well-known by conservation biologists and bird watchers for its high mosquito abundance and the presence of many wild birds [9]. Three cohorts of 5-day old sentinel chickens were set in open henhouses: 51 in P1 (16°16'49.1" North, 16°08'40.7" West), 73 in P2 (16°16'05.8" North, 16°07'59.5" West) and 55 chickens in P3 (16°16'30.1" North, 16°08'22.5" West) located at 1,290 m, 837 m, and 88 m from the river. For this purpose, 200 day-old chicks were purchased in a private hatchery and immediately vaccinated against Gumboro and Newcastle diseases. Seventeen of them were randomly sampled (23 October 2008) to assess their initial serological status against WNV. Chickens were identified with wing tags and placed in a box covered with a mosquito net to prevent exposure to mosquito bites before their transfer to Ross Bethio at day 5 (27 October 2008). During the survey, chicken deaths (34 in total) were not investigated for WNV infection because previous studies showed that chickens are unlikely to develop clinical disease [10]. Thus, we considered the observed mortality was not related to WNV.

Serological monitoring was done every 2 weeks from October 2008 to January 2009, *i.e.*, 6 times after the initial serological status assessment. Blood was sampled by puncture at the wing vein. Sera were collected after centrifugation (1,500 rpm during 5 min) and placed in 1.8 mL cryotubes stored at −20 °C until serological tests were done. Chicken sera were analyzed using an epitope-blocking enzyme-linked immunosorbent assay (ELISA), used in mammals as well as in wild and domestic birds [11,12]. This test uses a monoclonal antibody (3.1112G) that specifically binds to a unique NS1 polypeptide present only in WNV, and therefore has a high specificity for WNV. It is routinely used for WNV monitoring [13,14]. It detects anti-WN antibodies, including IgG and IgM, and can detect early reactions (less than 1 week post infection).

ELISA-positive sera were confirmed using microneutralization test (MNT) [15]. Heat-inactivated sera, serially diluted (1/5 to 1/3645) in Dulbecco’s modified Eagle’s medium (DMEM) were mixed with an equal volume (50 µL) of DMEM containing 100 50%-tissue-culture-infective doses (TCID_50_) of WNV, Is98 strain (kindly provided by P. Desprès, Institut Pasteur). Cell and virus (100 TCID_50_ of WNV) controls were added onto each plate. Moreover, 10^−1^, 10^−2^, 10^−3^, and 10^−4^ dilutions of the virus suspension were prepared for its back titration. After incubating the plates at 37 °C for 1.5 h, 2 × 10^4^ Vero cells in 100 µL of DMEM were added to every well. Plates were incubated at 37 °C for 3 days and read under microscope, looking for cytopathogenic effects. Results were validated if the following conditions were fulfilled: (i) absence of infected cells in the cell controls; (ii) presence of infected cells in the virus controls; (iii) virus titre comprised between 75 and 125 TCID_50_ per well; (iv) no protective effect visualized with the negative reference serum, e.g., every well with the negative reference sera was infected; (v) the positive reference serum protected Vero cells from infection, and the average neutralizing antibody titre for this positive reference serum was 90. A serum was considered as negative if cells were found infected at any serum concentration. It was considered as positive if cells were protected with the first serum dilution; its titre was calculated as the inverse of the latest dilution at which cells were protected.

We have adopted a discrete-time approach for data analysis [16]. For each chicken cohort, we counted the number of seroconversions occurring during each time interval defined by the sampling dates. To get the period-specific incidence rate, this number was divided by the corrected cohort size, defined as the number of chickens still present and seronegative at the beginning of the time interval, minus half of chickens lost-to-follow-up during this time interval (*i.e.*, before the next sampling date). To compare the cohorts with respect to the observed incidence rates, we performed a Fisher’s exact test on the table obtained after taking the geometric mean of the numbers at risk for each time interval, and the number of seroconversions over the whole study period. The null hypothesis was that the distribution of seroconversions was the same in the different cohorts.

The entomological survey consisted in monthly trapping during three consecutive nights in the three henhouses used for the chicken cohorts, and in a single horse-baited trap located at the same site as the P1 cohort (≈50 m away). This trap was a steel cage (2.5 × 1.5 × 2 m) containing a horse and covered with a mosquito netting (3.5 × 4 × 2.5 m) hanging at 15 cm above the ground, thus allowing mosquitoes to enter into the trap. This trap model has been used to study the potential vectors of WNV in southern France [17]. The chicken-baited trap was a henhouse (3 × 2 × 1.8 m) with screened openings on three sides (front, left and right) allowing chicken access for mosquitoes. It was covered with a mosquito net (4 × 3 × 2.5 m) hanging at 15 cm above the ground. Mosquito traps were set overnight from 6 pm to 6 am. Mosquitoes were collected inside the traps by aspiration. They were identified using the morphological keys of Edwards for the Culicinae subfamily [18] and Diagne *et al*., for the Anophelinae subfamily [19]. The engorged females were counted and separated from unfed and males and then stored at −70 °C for further studies. Trap catch for each mosquito species was assessed as the mean nightly number per trap (ANT). Chi-squared and Fisher tests were used to compare counts between groups. Non-parametric tests were used to compare ANTs: Wilcoxon test (two groups) and Kruskal-Wallis (>2 groups).

## 3. Results

### 3.1. Sentinel Serology

The WNV epitope blocking ELISA was performed on 923 chicken sera. Serological incidence rates are shown in Table 1. There were no positive sera among the 17 randomly-selected chickens at day 1, as well as in any of the 3 cohorts at day 15 (first interval, I_1_). A total of 29 samples tested ELISA-positive sera for WNV and were confirmed by MNT. All seroconversions occurred from 10 November to 10 December 2008 (*I_2_‒I_3_*). The overall serological incidence rate during the study period was 4.6% with 95% confidence interval (0.01; 0.08). The highest cohort-specific incidence (8.5%) was observed in cohort P3, close to the river. Cohort-P2 chickens, located at approximately 800 m from the river, had an overall incidence of 4.8%. Cohort-P1 chickens, (1,300 m from the river) remained free of WNV infection. These cohort-specific incidence rates were not significantly different according to a Fisher’s exact test (*p* = 0.13).

**Table 1:** Incidence of seroconversion (%) of WNV (and sample size) in three chicken cohorts placed in Ross Bethio (Senegal) from October 2008 to January 2009. Indices 1 to 6 indicate successive 2-week intervals starting at the end of October 2008. The overall incidence rate is *I_T_*.

Cohort	*I*_1_	*I*_2_	*I*_3_	*I*_4_	*I*_5_	*I*_6_	*I_T_*
P1	0.0 (0/51)	0.0 (0/47)	0.0 (0/42)	0.0 (0/41)	0.0 (0/40)	0.0 (0/40)	0.0 (0/43)
P2	0.0 (0/73)	1.4 (1/69)	3.2 (2/63)	0.0 (0/60)	0.0 (0/57)	0.0 (0/52)	4.8 (3/62)
P3	0.0 (0/55)	5.7 (3/53)	2.1 (1/48)	0.0 (0/44)	0.0 (0/42)	0.0 (0/42)	8.5 (4/47)

### 3.2. Entomological Survey

In total 10,192 mosquitoes belonging to eight species in three genera were caught during 51 trap-nights from October 2008 to January 2009 for chicken-baited traps, and from September 2008 to January 2009 for the horse-baited trap. *Culex* species accounted for 94% (*n* = 9,556) of total collection. The dominant species were *Culex neavei* with 51.2% (*n* = 5,218) of total, and *Culex tritaeniorhynchus* with 39.5% (*n* = 4,023) of total (Table 2).

**Table 2:** Mean nightly mosquito number per trap ± standard deviation in horse-baited and chicken-baited traps in survey sites from September 2008 to January 2009, Ross Bethio (Senegal); *n* is the number of trap-nights.

Mosquito species	Horse-baited trap (*n* = 15)	Chicken-baited traps			Total (%)
		P1 (*n* = 12)	P2 (*n* = 12)	P3 (*n* = 12)	
*Culex neavei*	176.7 ± 275.5	23.7 ± 15.1	44.4 ± 29.9	145.9 ± 133.1	5,218 (51.20)
*Culex tritaeniorhynchus*	257.4 ± 485.0	4.8 ± 8.3	2.5 ± 2.7	6.3 ± 4.3	4,023 (39.47)
*Mansonia uniformis*	2.2 ± 3.2	0.0	0.1 ± 0.3	1.0 ± 1.2	46 (0.45)
*Aedomyia africana*	0.1 ± 0.3	0.0	0.0	0.0	1 (0.01)
*Anopheles rufipes*	0.1 ± 0.3	0.0	0.0	0.0	1 (0.01)
*Anopheles pharoensis*	29.8 ± 44.3	0.4 ± 0.9	0.1 ± 0.3	1.7 ± 2.1	473 (4.64)
*Anopheles ziemanni*	7.1 ± 8.7	0.2 ± 0.4	0.0	0.5 ± 1.2	115 (1.13)
*Culex poicilipes*	20.1 ± 26.3	0.1 ± 0.3	0.1 ± 0.3	0.9 ± 1.3	315 (3.09)
Total					10,192

In chicken-baited traps, *Cx. neavei* was more abundant than *Cx. tritaeniorhynchus*. Moreover, collection decreased for the 2 species when the distance increased from Grand Lampsar River (P1 compared to P2 and P3: *p* = 0.05; *p* = 0.01) (Table 2). For *Cx. tritaeniorhynchus*, the ANTs were significantly different between P2 and P3, *p* = 0.025.

**Figure 1:**
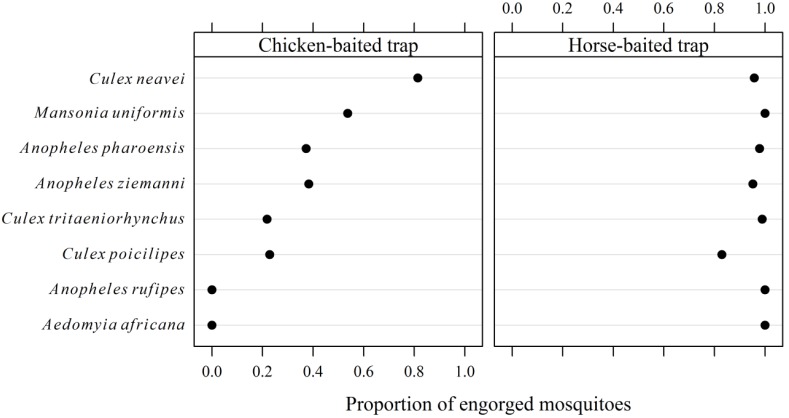
Engorgement rate of mosquitoes caught in chicken- and horse-baited traps in Ross Bethio (Senegal), October 2008 to January 2009.

A comparison of mosquito ANT in chicken- and horse-baited traps showed that *Cx. tritaeniorhynchus* was more mammophilic than ornithophilic, while *Cx. neavei* was attracted by both horse and birds (Table 2). In addition, all mosquito species found in the horse-baited trap showed a high engorgement rate (>80%), while only *Cx. neavei* and *Mansonia uniformis* showed a high engorgement rate in chicken-baited traps (circa 50%). Only 23% of *Cx. tritaeniorhynchus* had taken a blood meal in chicken-baited traps (Figure 1).

**Figure 2:**
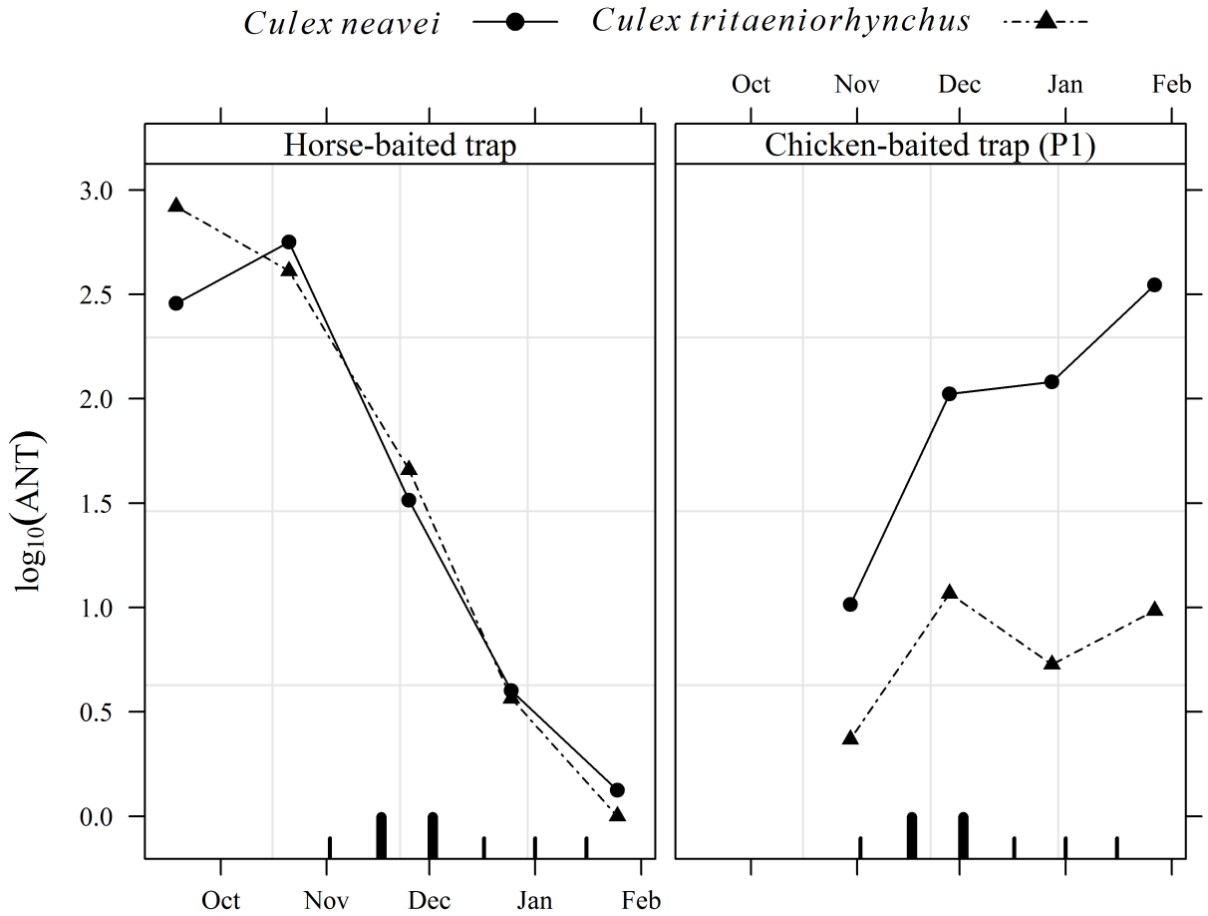
Mean nightly number of potential WNV mosquito vectors (broken curves) and serological incidence of WNV in a cohort of sentinel chickens (P1), from October 2008 to January 2009 in Ross Bethio, Senegal. Inner ticks on the time axis are mid-sampling intervals in sentinel chickens; longer and wider ticks are those where WNV seroconversions were observed in sentinel chickens.

ANT was similar for *Cx. neavei* and *Cx. tritaeniorhynchus* in the horse-baited trap (Figure 2). However, *Cx. neavei* was much more abundant than *Cx. tritaeniorhynchus* in the chicken-baited trap. Also, WNV transmission to sentinel chickens occurred during a period when a 10-fold increase of *Cx. neavei* ANT was observed.

## 4. Discussion

WNV was previously isolated in Senegal from several mosquito species [20,21,22]. Serological evidences of its transmission were also obtained in sentinel chickens [19], wild birds [23], horses [24,25], and humans [26]. During the same time period and study areas used in this study, a survey in 570 adult horses revealed a serological prevalence rate of 93% [27]. 

Results suggested that WNV was amplified through a bird-mosquito cycle in Ross Bethio, during the survey period. Other Flaviviruses have been described in the past in the same or neighbor regions: Usutu, Bagaza, Saboya and Wesselsbron viruses [21,22,28]. However, their presence was not investigated in the current study.

Sentinel chickens are widely used to monitor WNV activity [10,23,29,30]. In this study, WNV transmission was detected rather late during the rainy/flooding season: November and December 2008, a time period dominated by the proliferation of potential mosquito vectors in the region [7].

WNV incidence in sentinel chickens also coincided with increasing *Cx. neavei* ANT in chicken-baited traps. This species was the main vector incriminated in WNV transmission in the region [7]. However, its involvement was limited in time because *Cx. neavei* populations faded out by February in favor of *Cx. poicilipes*, according to a monthly mosquito population dynamics study implemented in the same area from September 2005 to April 2007 (Fall *et al*., unpublished data). However, the role of these two mosquito species in WNV transmission should be further investigated.

In wetland areas where malaria is a frequent disease, there is a high risk of misdiagnosing mild or even severe WN human cases. Such insidious events have been detected in Guinea and Madagascar, for instance [31,32]. Moreover, clinical cases may occur in immunologically naïve travelers visiting Senegal or other similar ecosystems [33,34,35]. In addition, most equids in the area have WNV antibodies, making it unlikely to see clinical signs in these animals [25,27]. However, the systematic investigation of encephalitis cases in horses (especially foals) and humans would be useful to detect the emergence of neuro-invasive strains of WNV. With this respect, training of veterinary and human health officers would help achieve this goal.

## 5. Conclusions

Our results highlighted WNV transmission in sentinel chickens in the Senegal River Delta. Moreover, we identified mosquito vectors possibly involved in WNV transmission. Because this area is frequented by many foreign people immunologically naïve with respect to WNV, these findings have a public-health interest. Moreover, WNV monitoring in sentinel chickens and in targeted mosquito populations, as well as reinforced passive surveillance of clinical cases in horses and humans, might provide useful information regarding circulating WNV and the possible emergence of more pathogenic strains.
